# Zn(II) and Cd(II) Complexes of AMT1/MAC1 Homologous Cys/His-Rich Domains: So Similar yet So Different

**DOI:** 10.1021/acs.inorgchem.2c02080

**Published:** 2022-08-31

**Authors:** Anna Rola, Paulina Potok, Magdalena Mos, Elżbieta Gumienna-Kontecka, Sławomir Potocki

**Affiliations:** † Faculty of Chemistry, University of Wroclaw, 14 Joliot-Curie Street, 50-383 Wroclaw, Poland; ‡ WMG, International Manufacturing Centre, University of Warwick, Coventry CV4 7AL, United Kingdom

## Abstract

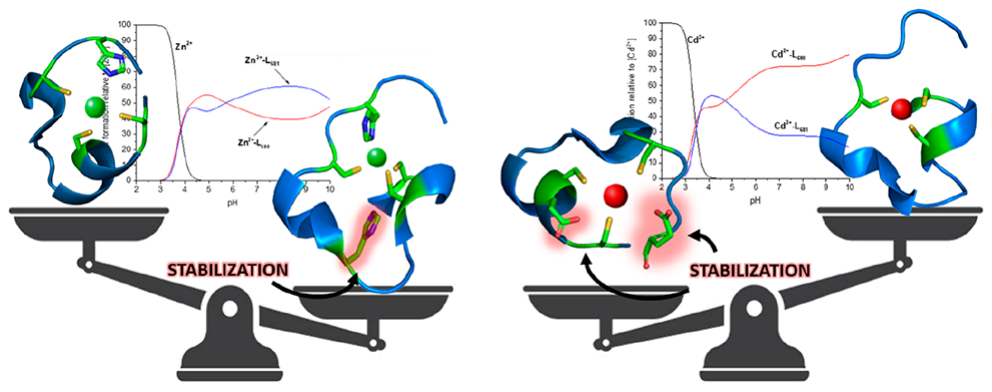

Infections caused by *Candida* species are becoming seriously dangerous and difficult to cure due to their sophisticated mechanisms of resistance. The host organism defends itself from the invader, e.g., by increasing the concentration of metal ions. Therefore, there is a need to understand the overall mechanisms of metal homeostasis in *Candida* species. One of them is associated with AMT1, an important virulence factor derived from *Candida glabrata*, and another with MAC1, present in *Candida albicans*. Both of the proteins possess a homologous Cys/His-rich domain. In our studies, we have chosen two model peptides, L680 (Ac-_10_ACMECVRGHRSSSCKHHE_27_-NH_2_, MAC1, *Candida albicans*) and L681 (Ac-_10_ACDSCIKSHKAAQCEHNDR_28_-NH_2_, AMT1, *Candida glabrata*), to analyze and compare the properties of their complexes with Zn(II) and Cd(II). We studied the stoichiometry, thermodynamic stability, and spectroscopic parameters of the complexes in a wide pH range. When competing for the metal ion in the equimolar mixture of two ligands and Cd(II)/Zn(II), L680 forms more stable complexes with Cd(II) while L681 forms more stable complexes with Zn(II) in a wide pH range. Interestingly, a Glu residue was responsible for the additional stability of Cd(II)-L680. Despite a number of scientific reports suggesting Cd(II) as an efficient surrogate of Zn(II), we showed significant differences between the Zn(II) and Cd(II) complexes of the studied peptides.

## Abstract 2

*Candida* species developed sophisticated pathways of metal homeostasis. One of them is associated with AMT1 (*Candida glabrata*), and another with MAC1 (*Candida albicans*). Both of the proteins possess a homologous Cys3His-type domain. We studied two model peptides, L680 (Ac-_10_ACMECVRGHRSSSCKHHE_27_-NH_2_, MAC1, *C. albicans*) and L681 (Ac-_10_ACDSCIKSHKAAQCEHNDR_28_-NH_2_, AMT1, *C. glabrata*), and analyzed their properties as ligands for Zn(II) and Cd(II). Interestingly, a Glu residue was responsible for the additional stability of Cd(II)-L680 compared to that of Cd(II)-L681.

## Introduction

An alarmingly growing number of multidrug resistant (MDR) *Candida* species, e.g., *Candida albicans* and *Candida glabrata*, attack immunocompromised and immunocompetent patients.^1−4^ The mechanism of antifungal resistance, especially azole resistance, is based on overexpression of multidrug efflux pumps, modifications of target proteins, and adjustments in the composition of the membrane sterol.^5^ The problem of MDR *Candida* species has prompted the scientific community to look for alternative methods of treatment, based on, e.g., metal ions. The very first step is to understand, on a molecular level, the mechanisms of metal homeostasis in *Candida* species. They are commensal in nature and live in mucous membranes and the skin of healthy host organisms.^6^ Disturbances to this delicate balance, e.g., alterations in the local environment (including pH variations or nutritional changes), use of antifungal antibiotics, or variations in the immune system (infections/immunosuppressant therapy), lead to the rapid proliferation and invasion of *C. albicans*.^7−10^ These include mucosal and skin infections, such as vaginal yeast infections, thrush, diaper rash, and more serious hematogenously disseminated infections with high mortality rates (approaching even 47%).^7^*C. albicans*, one of the *Candida* species, is the most common reason for hospital-acquired infections. It is responsible for 40% of bloodstream infections in clinical settings and 15% of all sepsis cases.^11−15^ Furthermore, almost 50% of candidemia cases in the United States are associated with *C. albicans* while *C. glabrata* is responsible for 25% of infections.^16,17^ Similarly to *C. albicans*, *C. glabrata* is strongly resistant to fluconazole, itraconazole, voriconazole, and posaconazole.^18,19^

The host organism defends itself from the invader, e.g., by increasing the concentration of metal ions such as zinc and copper.^20^*Candida* species developed complicated pathways of maintaining metal homeostasis during the virulence process. One of them is controlled by AMT1 (metal-activated transcriptional activator protein 1, *C. glabrata*). This particular protein is activated in the presence of copper or silver ions and controls the expression of genes responsible for the production of metallothioneins.^21^ AMT1 possesses an N-terminal Cys/His-rich domain, responsible for binding zinc ions and consisting of a series of amino acid residues responsible for that coordination (Cys-X_2_-Cys-X_8_-Cys-X-His, where X means the next amino acid residue and the number after it means the number of following amino acid residues).^22^ We identified the homologous domain in the *C. albicans* MAC1 protein (metal-binding activator 1).^22^ What we found interesting is that “the nature chose” the homologue sequences for two fungal metal-interacting proteins in two different *Candida* species, despite the fact that the role of the proteins is not exactly the same. MAC1 activates under low-copper conditions and induces transcription of the CTR1 copper transporter.^23−25^ The mutant lacking MAC1 displays slow growth on low-copper medium and low-iron medium and is hypersensitive to exposure to heat and/or cadmium.^23−25^ Interestingly, we determined that particular Cys3His-type motifs are likely to be found especially in fungal proteins.^26−30^ The sequences of model peptides of the AMT1 and MAC1 Cys/His-rich domain are partially different from each other (Table 1).

**Table 1:** Sequences of AMT1 (*C. glabrata*) and MAC1 (*C. albicans*) Domains with High Affinity for Zinc Ions

MAC1 *C. albicans*	L680, Ac-_10_ACMECVRGHRSSSCKHHE_27_-NH_2_
AMT1 *C. glabrata*	L681, Ac-_10_ACDSCIKSHKAAQCEHNDR_28_-NH_2_

Studies of the coordination chemistry of individual metal-binding domains can provide information regarding the importance of single amino acid residues in the stability of complexes with the zinc ions. Zinc is a crucial trace element for fungal organisms, as it occupies the structural and catalytic center of a wide variety of proteins. It is essential for the survival and virulence of yeast such as *Candida* in humans.^21^ Most of the zinc-binding proteins participate in biological processes related to transcriptional regulation of the cellular metabolic network. In addition, there are numerous zinc-binding enzymes involved in fungal virulence, including superoxide dismutases, alcohol dehydrogenase, and metalloproteases.^31^ On the contrary, cadmium is a relatively rare metal, which is known to be a potent toxicant to microorganisms such as fungi or bacteria.^32^ Its toxicity is a result of several similarities between cadmium and zinc ions.^33^ Both of them belong to the same group of the periodic table and have the same oxidation state (+2). That can lead to substitution of Zn(II) with Cd(II) in biological systems, especially proteins with a sulfur-dominated coordination sphere.^34^ Cd(II) is used as a substituent for the Zn(II) ion in zinc sites of metalloproteins.^35^ The isoelectronic nature of Cd(II) and Zn(II) (d^10^ outer electronic configurations) and the efficient nuclear magnetic resonance (NMR) properties of the ^113^Cd isotope have resulted in its use as a NMR spectroscopic probe, e.g., for zinc proteins and other model compounds.^36^

The study was focused on understanding bioinorganic and coordination chemistry of Cys/His-rich MAC1/AMT1 domains as ligands for Zn(II) and Cd(II). The main purpose was to describe the coordination of Zn(II) for the studied peptides that are naturally dedicated to this metal ion, but the second, no less important goal was to check whether cadmium is an efficient substitute in these particular systems for zinc. For this purpose, the stoichiometry, stability, and metal-binding sites of formed complexes of model peptides were investigated (Table 1). We wanted to investigate the similarities and differences of two of such independent (different origin, fungal species) domains that could help to explain (A) the coordination properties of the MAC1 Cys/His-rich domain and (B) the impact of adjacent to Cys3His2 motif amino acid residues on their Zn(II)/Cd(II) complex properties. We investigated the behavior of model peptides and established complex species present in solution in a wide pH range of 2–11. Our study provides interesting information concerning bioinorganic and coordination chemistry of AMT1 and MAC1. The set of data obtained in this study may be an input to understand the metal homeostasis in *Candida* species.

## Experimental Section

### Peptide Synthesis and Purification

All peptides were purchased from KareBay Biochem, Inc., with a certified purity: L680, 98.27%; L681, 98.08%. The identity of ligands was confirmed by mass spectrometry. The purity was examined by potentiometric titrations with the use of the Gran method.^37^ The solutions of metal ions were prepared using ZnClO_4_ and CdClO_4_ (POCh, high-performance liquid chromatography grade). The metal salts were dissolved in doubly distilled and filtered water. The concentration of a stock solution was periodically checked via ICP-MS. The solution of 4 × 10^–3^ M HClO_4_ (Merck) was used to prepare all samples of the peptides. The ionic strength was adjusted to 0.1 mol dm^–3^ by adding KClO_4_ (Merck).

### Mass Spectrometry Measurements

All of the mass spectra were recorded for the mixtures of peptides and metal ions dissolved in a MeOH/H_2_O solution (1:1); the M(II):L molar ratio equaled 1:1. The ligand concentration was 1 × 10^–4^ M. Two types of instruments were used in this experiment, both operated in positive ion mode.

Mass spectra of Zn(II)-L systems were recorded using a Fourier transform ion cyclotron resonance (FT-ICR) Apex-Qe Ultra 7T appliance (Bruker Daltonics, Bremen, Germany) equipped with an Apollo II ESI (electrospray ionization) source with an ion funnel. The following parameters were used: drying gas, N_2_; flow rate, 4 L/min; *m*/*z* range, 1000–2200; internal capillary temperature, 200 °C; voltage, 4500 V. The Tunemix mixture (Bruker Daltonics) was used for an instrument calibration using a quadratic method. The mass spectra were analyzed using Compass DataAnalysis 4.0 (Bruker Daltonics).

Mass spectra of Cd(II)-L systems were recorded with using a LC-MS qTOF 9030 Shimadzu (Kyoto, Japan) mass spectrometer equipped with a standard electrospray ionization source and LC system. The appliance was calibrated with the sodium iodate (Merck, Darmstadt, Germany) with a quadratic method. The following measurement parameters were used: volume of injection, 1 μL; *m*/*z* scan range, 100–1000; nebulizing gas flow, 3 L min^–1^; drying gas, nitrogen; flow rate, 4.0 L min^–1^; interface temperature, 200 °C; heat block temperature, 300 °C; DL temperature, 250 °C; potential between the spray needle and the orifice, 4.0 kV. Lab solution software was used for the processing and analysis of MS spectra. The data were assessed with ACD/Spectrus Processor 2021 2.0 software.

### Potentiometric Measurements

Stability constants for the proton as well as metal complexes were calculated from the pH-metric titration curves. All experiments were carried out under an argon atmosphere to protect the sample from the appearance of carbonates. The other parameters were as follows: temperature, 298 K; pH range, 2.5–11; solvent, 4 mM HClO_4_ water solution with an ionic strength of 0.1 M NaClO_4_. The potentiometric measurements were carried out using a pH electrode InLab Semi-Micro instrument (Mettler Toledo), and a Dosimat 665 Methrom titrator connected to a Methrom 691 pH-meter. The calibration of the electrode in terms of hydrogen concentration was achieved by titrating HClO_4_ with carbonate-free NaOH under the same experimental conditions described above. The purities and exact concentrations of the ligand solutions were established by the Gran method. The peptide concentration was 0.5 mM. The metal:ligand molar ratio equaled 1:1.

The potentiometric data were processed with HYPERQUAD 2006.^38^ Reported log β values refer to the overall equilibria:
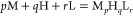
1
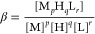
2where charges are omitted for the clarity and log *K*_step_ values refer to the protonation process:
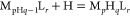
3(charges omitted; *p* might also be 0). Standard deviations were calculated with HYPERQUAD 2006 and refer to random errors only. The speciation and competition diagrams were computed with the HYSS program.^39^

### NMR Measurements

Nuclear magnetic resonance (NMR) experiments were carried out at 14.1 T on a Bruker Avance III 600 MHz instrument equipped with a Silicon Graphics workstation at controlled temperatures (±0.1 K). The residual water signal was suppressed by excitation sculpting, using a selective square pulse on water 2 ms long. The solutions of analyzed peptides were prepared in a 90% H_2_O/10% D_2_O (99.95% from Merck) mixture. The assignment was accomplished with two-dimensional (2D) ^1^H–^1^H total correlation spectroscopy (TOCSY) and nuclear Overhauser effect spectroscopy (NOESY) experiments, performed with standard pulse sequences. Spectral processing and analysis were carried out using Bruker TOPSPIN 2.1, Cara, and MestreNova software. Samples of analyzed complexes were prepared by adding a metal ion to an acidic solution of 0.8 mM ligand (pH 7.4), with a total sample volume of 600 μL.

### Ultraviolet–Visible (UV–vis) Spectroscopy Measurements

The absorption spectra in the UV–vis region of the cadmium complexes were recorded using a Cary 300 Bio spectrophotometer, in the 800–250 nm range at 298 K using a total volume of 2.8 mL. The instrument parameters were as follows: number of accumulations, 3; scanning speed, 500 nm/min; data pitch, 0.5 nm. The concentration of the ligands was 4 × 10^–4^ M for the metal complexes. Ligand:metal molar ratios were 1:1. Data processing was achieved using Origin version 9.0.

## Results and Discussion

MAC1 and AMT1 are significant virulence factors. Their main function is to regulate metal ion concentration in *Candia* species. To describe the coordination chemistry of such metal–protein complexes, our study focused on the properties of domains with a high affinity for Zn(II) [or Cd(II)]. We wanted to determine how many Zn(II)/Cd(II) ions can interact with one molecule of model peptides, which residues are binding sites for Zn(II)/Cd(II) ions, and how the stability of complexes changes with an increase in pH. The results of this study bring us closer to a full understanding of the MAC1/AMT1 coordination chemistry and the mechanism of metal homeostasis controlled by them in *Candida* species.

### Protonation Equilibria of the Ac-_10_ACMECVRGHRSSSCKHHE_27_-NH_2_ (L680) and Ac-_10_ACDSCIKSHKAAQCEHNDR_28_-NH_2_ Peptides

The protonation constants of examined peptides and proposed assignments to the particular chemical groups are listed in Table 2. Charges of the species have been omitted to improve the clarity of the table. Ac-_10_ACMECVRGHRSSSCKHHE_27_-NH_2_ (L680) behaves like H_9_L acid, while Ac-_10_ACDSCIKSHKAAQCEHNDR_28_-NH_2_ (L681) like H_10_L acid in pH range of 2–11. The model peptides were protected at the N- and C-terminus. p*K*_a_ values calculated for the studied peptides are in line with the literature data of the deprotonation constants for peptides.^40^

**Table 2:** Potentiometric Data for Peptides Ac-_10_ACMECVRGHRSSSCKHHE_27_-NH_2_ (L680) and Ac-_10_ACDSCIKSHKAAQCEHNDR_28_-NH_2_ (L681) and Their Zn(II) and Cd(II) Complexes^a^

Ac-_10_ACMECVRGHRSSSCKHHE_27_-NH_2_ (L680)			Ac-_10_ACDSCIKSHKAAQCEHNDR_28_-NH_2_ (L681)		
species	log β*_jk_*^b^	p*K*_a_^c^	species	log β*_jk_*^b^	p*K*_a_^c^
HL	10.90 (1)	10.90 (Lys)	HL	10.83 (2)	10.83 (Lys)
H_2_L	20.59 (1)	9.69 (Cys)	H_2_L	21.16 (1)	10.33 (Lys)
H_3_L	29.27 (1)	8.68 (Cys)	H_3_L	30.58 (2)	9.42 (Cys)
H_4_L	37.27 (1)	8.00 (Cys)	H_4_L	39.26 (2)	8.68 (Cys)
H_5_L	44.03 (1)	6.76 (His)	H_5_L	47.27 (2)	8.01 (Cys)
H_6_L	50.33 (1)	6.30 (His)	H_6_L	54.01 (2)	6.74 (His)
H_7_L	55.75 (1)	5.41 (His)	H_7_L	59.98 (2)	5.96 (His)
H_8_L	59.74 (2)	4.00 (Glu)	H_8_L	64.16 (3)	4.19 (Glu)
H_9_L	62.89 (2)	3.15 (Glu)	H_9_L	67.76 (3)	3.61 (Asp)
			H_10_L	70.44 (3)	2.68 (Asp)

Zn(II) Complexes					
species	log β*_ijk_*^d^	p*K*_a_^e^	species	log β_*ijk*_^d^	p*K*_a_^e^
ZnH_5_L	49.60 (7)		ZnH_5_L	54.12 (2)	
ZnH_4_L	45.17 (4)	4.43	ZnH_4_L	–	
ZnH_3_L	40.36 (2)	4.81	ZnH_3_L	44.46 (1)	
ZnH_2_L	34.58 (2)	5.78	ZnH_2_L	38.21 (4)	6.25
ZnHL	27.83 (2)	6.75	ZnHL	27.87 (7)	10.34
ZnL	17.53 (4)	10.31	ZnL	17.35 (7)	10.52
ZnH_–1_L	6.99 (3)	10.54 (H_2_O)	ZnH_–1_L	6.46 (7)	10.90 (H_2_O)

Cd(II) Complexes					
species	log β*_ijk_*^d^	p*K*_a_^e^	species	log β*_ijk_*^d^	p*K*_a_^e^
CdH_5_L	50.62 (6)		CdH_5_L	55.35 (6)	
CdH_4_L	46.51 (4)	4.12	CdH_4_L	50.66 (7)	4.69
CdH_3_L	41.53 (5)	4.98	CdH_3_L	45.48 (7)	5.19
CdH_2_L	36.34 (3)	5.19	CdH_2_L	39.06 (8)	6.42
CdHL	29.90 (3)	6.44	CdHL	–	
CdL	19.75 (5)	10.04	CdL	18.81 (9)	

a The proposed assignments are given in parentheses.

b Protonation constants are presented as cumulative log β_*jk*_ values. Standard deviations of the last digits are given in parentheses, at the values obtained directly from the experiment. L stands for a peptide with acid–base active groups. β(H*_j_*L_*k*_) = [H*_j_*L*_k_*]/([H]^*j*^[L]^*k*^), in which [L] is the concentration of the fully deprotonated peptide.

c p*K*_a_ = log β(H*_j_*L_*k*_) – log β(H_*j*–1_L*_k_*).

d Zn(II) and Cd(II) stability constants are presented as cumulative log β*_ijk_* values. L stands for a fully deprotonated peptide ligand that binds Cd(II). Standard deviations of the last digits are given in parentheses, at the values obtained directly from the experiment. β(M_*i*_H*_j_*L*_k_*) = [M*_i_*H*_j_*L*_k_*]/([M]^*i*^[H]^*j*^[L]*^k^*), where [L] is the concentration of the fully deprotonated peptide.

e p*K*_a_ = log β(M*_i_*H*_j_*L*_k_*) – log β(M_*i*_H_*j*–1_L*_k_*).

### Metal Complexes

**Figure 1:**
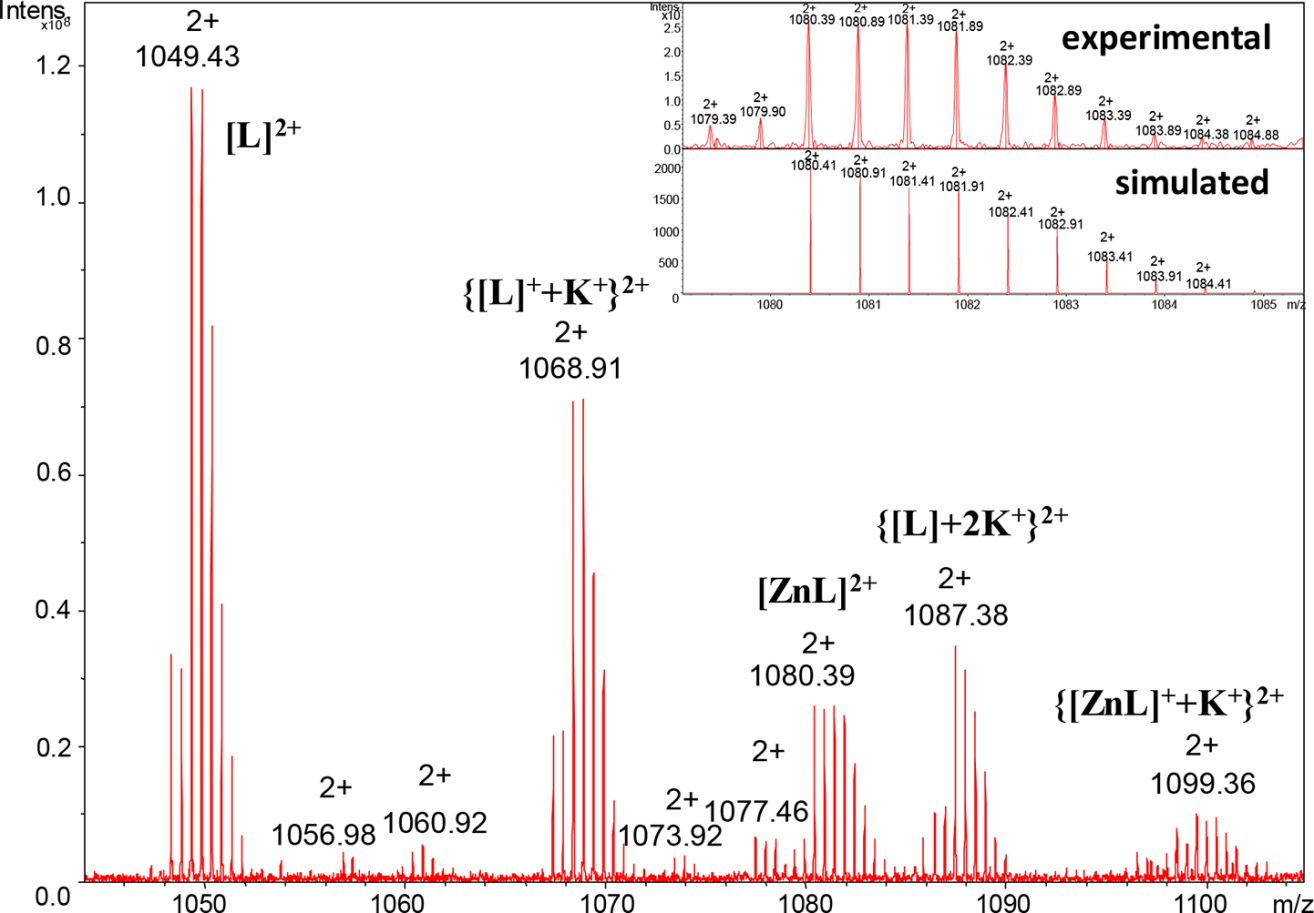
ESI-MS spectrum of a system composed of the Ac-_10_ACMECVRGHRSSSCKHHE_27_ ligand (L680) and Zn(II) ions in the range of *m*/*z* 1044–1150 at pH 7.4 (1:1 M:L). In the top right corner, the simulated and experimental isotopic distribution spectra with a peak at *m*/*z* 1080.39 are presented.

**Figure 2:**
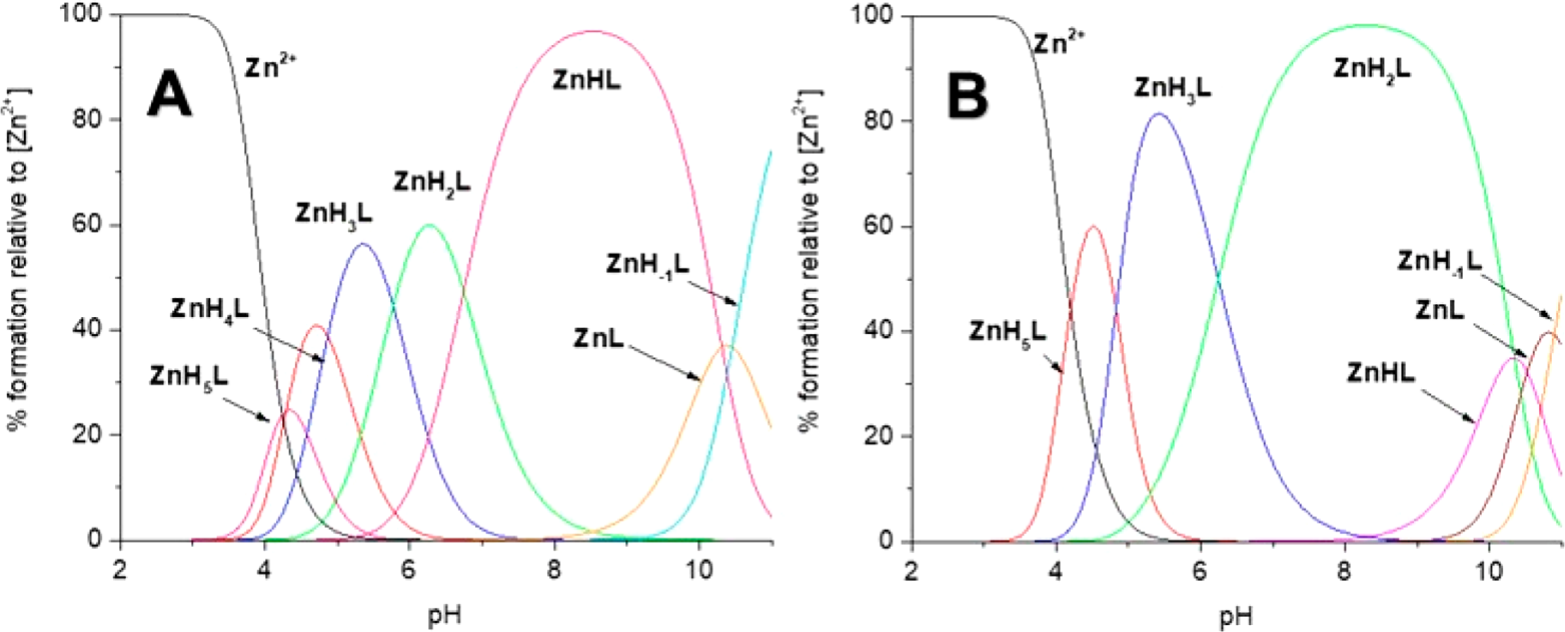
Distribution diagram of complex forms in the studied Zn(II)-L systems: (A) Ac-_10_ACMECVRGHRSSSCKHHE_27_ and (B) Ac-_10_ACDSCIKSHKAAQCEHNDR_28_-NH_2_ at a M:L ratio of 1:1 in the pH range of 2–11.

**Figure 3:**
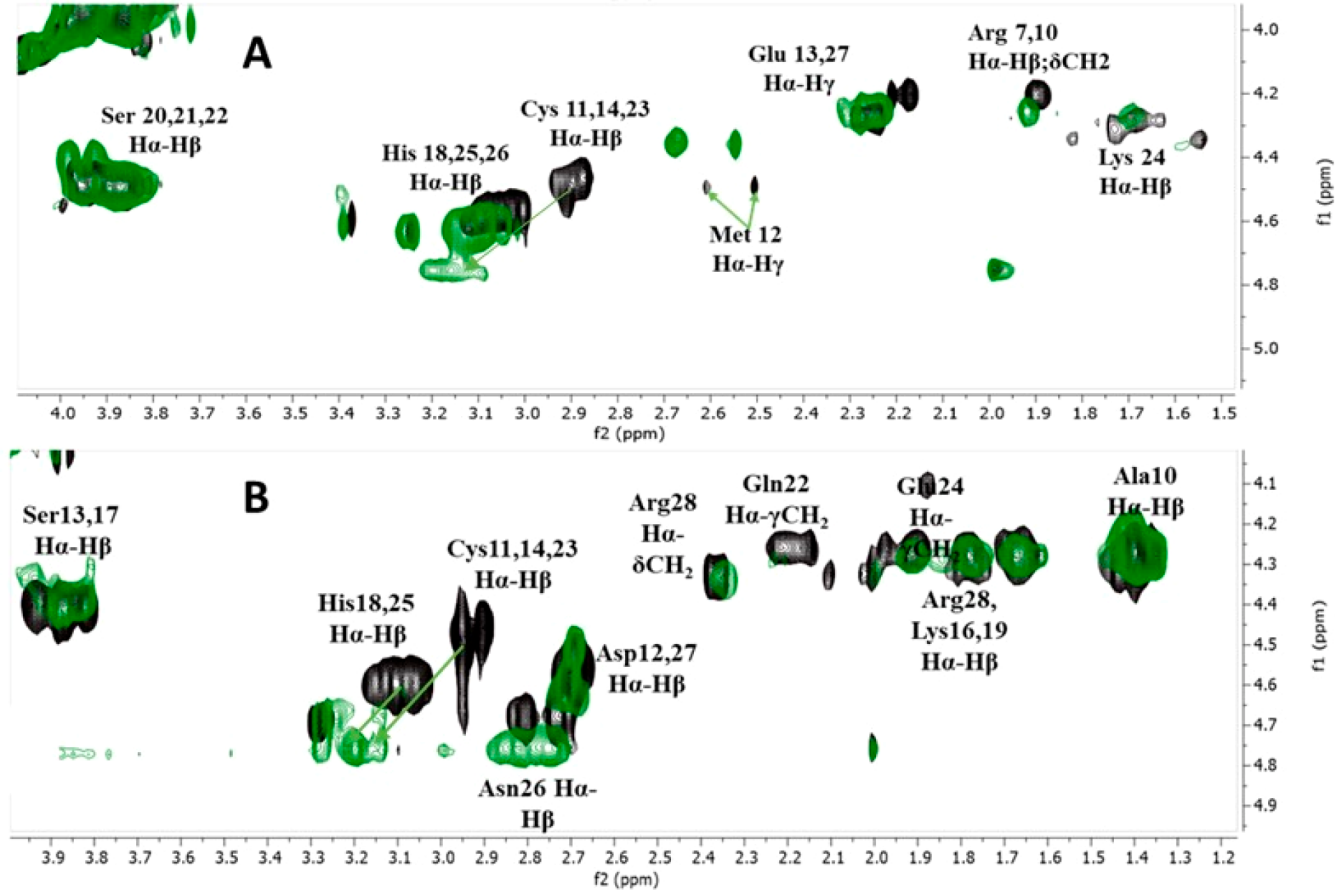
Fragment of ^1^H–^1^H TOCSY NMR spectra of the peptide (black) and the Zn(II) complex (green) with (A) Ac-_10_ACMECVRGHRSSSCKHHE_27_-NH_2_ and (B) Ac-_10_ACDSCIKSHKAAQCEHNDR_28_-NH_2_ at pH 7.4, a M:L ratio of 1:1, and 298 K.

**Figure 4:**
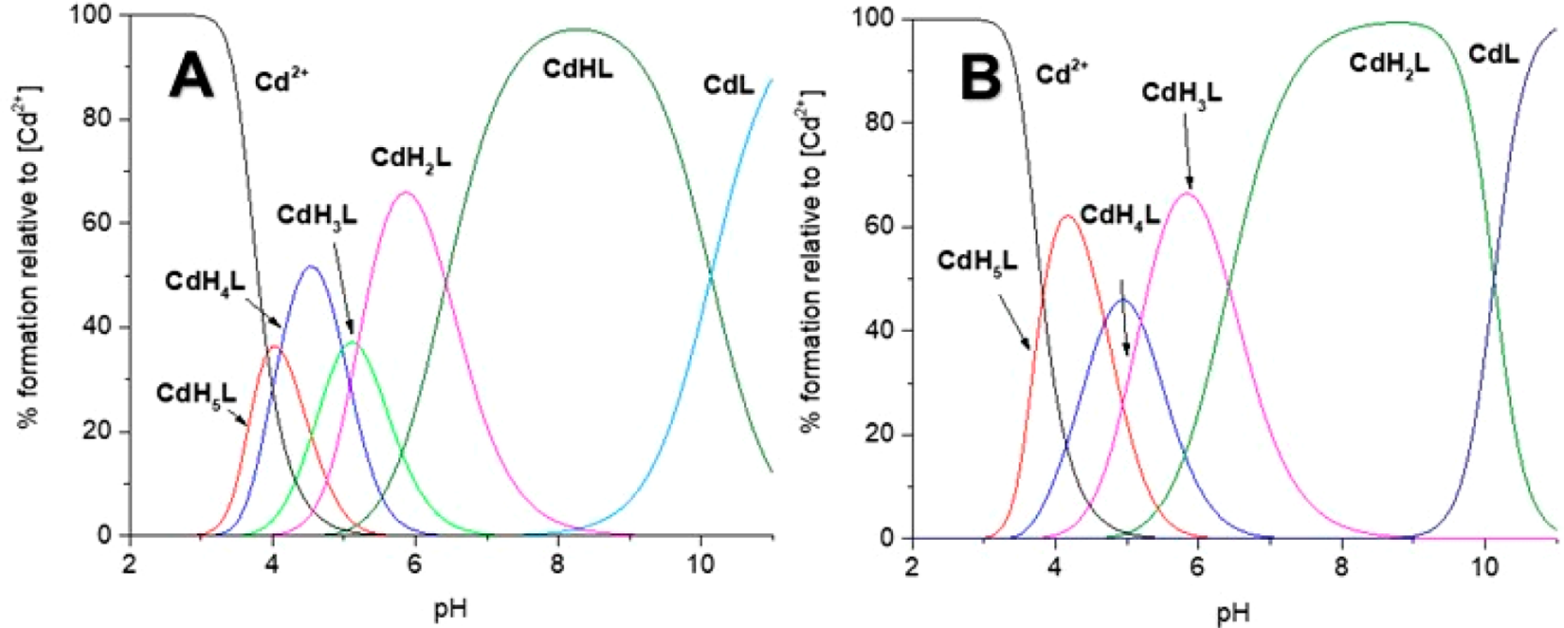
Distribution diagram of complex forms in the studied Cd(II)–L systems: (A) Ac-_10_ACMECVRGHRSSSCKHHE_27_ and (B) Ac-_10_ACDSCIKSHKAAQCEHNDR_28_-NH_2_ at a M:L ratio of 1:1 in the pH range of 2–11.

**Figure 5:**
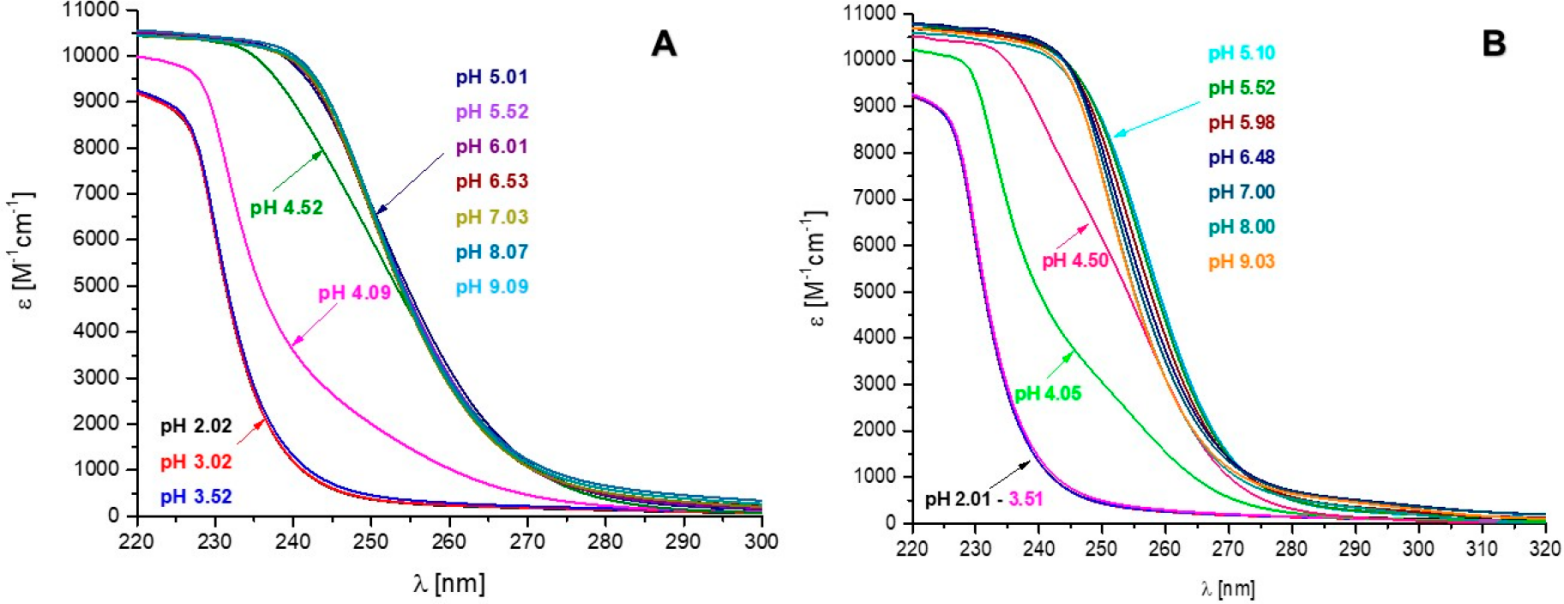
UV–vis spectrum of Cd(II) complexes with (A) Ac-_10_ACMECVRGHRSSSCKHHE_27_-NH_2_ and (B) Ac-_10_ACDSCIKSHKAAQCEHNDR_28_-NH_2_ peptide over the pH range of 2–9 at 298 K, a metal:ligand ratio of 1:1; and 2.5 × 10^–4^ M Cd(II).

**Figure 6:**
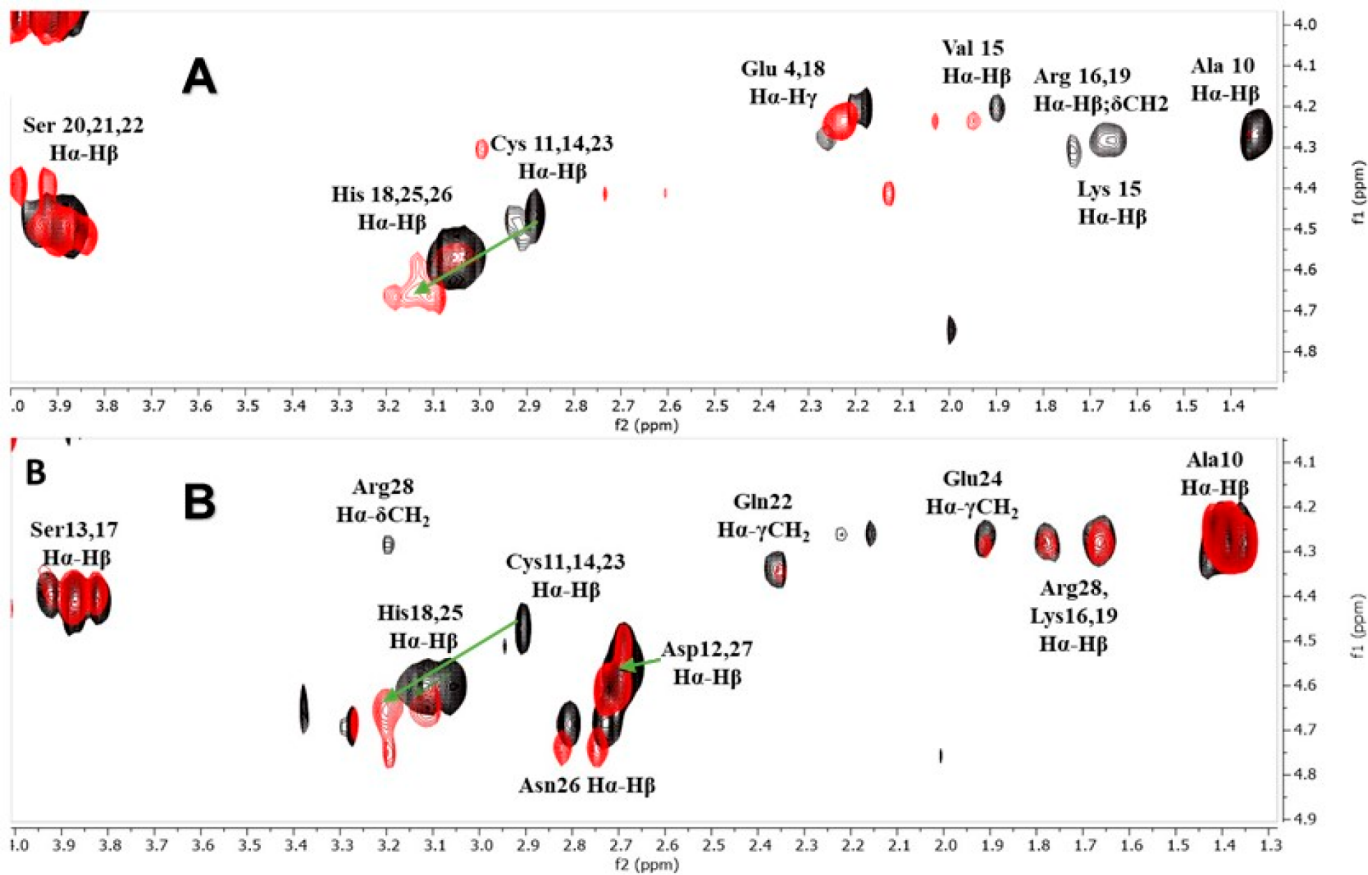
Fragment of ^1^H–^1^H TOCSY NMR spectra of the peptide (black) and the Cd(II) complex (red) with the peptide: (A) Ac-_10_ACMECVRGHRSSSCKHHE_27_-NH_2_ and (B) Ac-_10_ACDSCIKSHKAAQCEHNDR_28_-NH_2_ at pH 7.4, a M:L ratio of 1:1, and 298 K.

The presence of metal [Zn(II)/Cd(II)] complexes with studied peptides was confirmed by a variety of analytical methods. Signals in the mass spectra have been assigned to ions of ligands or their metal complexes. The assignment of ESI-MS peaks was based on the comparison between the calculated and experimental *m*/*z* values and their isotopic patterns. MS was also used to characterize the stoichiometry of the formed metal complexes. The results of potentiometric titrations were used to establish the stability of complexes. UV–vis and NMR spectroscopy indicated the metal-binding sites and helped to explain potentiometric results. Stability constants for examined complexes are listed in Table 2; the distribution diagrams and MS/NMR/UV–vis spectra are presented in Figures 1–6 and the Supporting Information.

### Zn(II) Complexes

The mass spectrum of the Zn(II)–L680 (charge 2+) system is shown in Figure 1. The spectrum shows peaks corresponding to free ligand ion [L]^2+^ (*m*/*z* 1049.43; *z* = 2+) and an equimolar complex with Zn(II) ions [ZnL]^2+^ (*m*/*z* 1080.39; *z* = 2+). In addition, we have observed potassium ({[L]^+^+K^+^}^2+^, *m*/*z* 1068.91; *z* = 2+), two potassium ({[L]+2K^+^}^2+^, *m*/*z* 1087.38; *z* = 2+), and potassium to the Zn(II) complex ion ({[ZnL]^+^+K^+^}^2+^, *m*/*z* 1099.36; *z* = 2+) adducts. In the top right corner of Figure 1, an isotopic distribution of the mononuclear complex ion [ZnL]^2+^ (*m*/*z* 1080.39; *z* = 2+) is shown. The 1:1 metal–ligand interaction is confirmed by potentiometric calculations. The mass spectra of the Zn(II)–ligand systems with ligand L681 are shown in Figure S1. In the spectrum, we can observe the [L]^2+^ signal corresponding to the doubly charged ligand ion as well as [ZnL]^2+^ signal corresponding to the doubly charged ion of the mononuclear Zn(II)–ligand complex. In the spectrum, we can identify signals corresponding to the potassium adduct ions of the examined ligand and its Zn(II) complex, such as {[L]^+^+K^+^}^2+^ and {[ZnL]^+^+K^+^}^2+^. Under the conditions prevailing inside the ion source, ligand L681 tends to form an adduct with a water molecule forming {[ZnL]^2+^+H_2_O}^2+^ ion.

### Coordination Mode and Thermodynamics

The potentiometric titrations of the Zn(II)–L680 (Ac-_10_ACMECVRGHRSSSCKHHE_27_-NH_2_) system showed the existence of seven Zn(II) complex forms in the pH range of 2–11: ZnH_5_L, ZnH_4_L, ZnH_3_L, ZnH_2_L, ZnHL, ZnL, and ZnH_–1_L (Table 2 and Figure 2A). The first three detected complex species were ZnH_5_L and ZnH_4_L starting to form at pH 3.5–4.0. Most probably, they come from the deprotonation of two and three Cys residues, respectively. In these two forms also, Glu residues are deprotonated. A p*K*_a_ value of 4.43 is significantly reduced compared to p*K*_a_ values of 9.69, 8.68, and 8.00 for Cys residues in the free ligand, suggesting the presence of three Cys side chains in the coordination sphere of Zn(II). Usually, Cys residues bind Zn(II) at pH ∼4.0–5.0.^41,42^ The next three detected species are ZnH_3_L, ZnH_2_L, and ZnHL, formed at pH ∼4.0, ∼4.5, and ∼5.5, respectively. The most significant form, ZnHL, reaches its maximum concentration at pH ∼8.5. Most probably, the three species come from the deprotonation of histidine’s side chains. The p*K*_a_ values of these steps equal 4.81, 5.78, and 6.75, respectively, which are not significantly reduced compared to p*K*_a_ values of 5.41, 6.30, and 6.76, respectively, of the His residues in free ligand. On the basis of only potentiometry results, it was not clear whether these histidine residues bind Zn(II); however, one of them could due to a larger difference in the p*K*_a_ in the free ligand (5.41) and complex (4.81). The formation of complex species and the involvement of His in Zn(II) binding were confirmed by NMR analysis. After the addition of 0.9 equiv of Zn(II) ions to L680 at pH 7.4 (physiologically relevant pH at which the ZnHL form dominates), selective chemical shift variations were detected by comparing H^1^–H^1^ TOCSY spectra recorded for apo and Zn(II)-bound forms (Figure 3A). The presence of Zn(II) ions caused the shift of overlaid Hα–Hβ His-18, -25, and -26 and larger shifts of Hα–Hβ Cys-11, -14, and -23 correlations in ^1^H–^1^H TOCSY spectra, which indicates that all cysteines are strongly involved in metal binding. It also shows that one histidine residue may indeed strongly interact with the Zn(II) ion or at least stabilize the structure of complex. It is in agreement with potentiometric results. The shift of Hα–Hγ Met-12 (non-metal-binding amino acid residue) is related to the close neighborhood of the metal ion. The next detected complex form is ZnL, formed at pH ∼8.0, and its maximum concentration occurs at pH 10.5. This formation is related to the deprotonated lysine residue (p*K*_a_ = 10.31), which is not involved in metal binding. ZnH_–1_L is the last calculated complex. It appears in the solution at pH 9.0. This formation is most probably related to the deprotonation of the water molecule coordinated to the Zn(II) ion.

The Zn(II)–Ac-_10_ACDSCIKSHKAAQCEHNDR_28_-NH_2_ system showed the existence of six complex forms at pH 2–11: ZnH_5_L, ZnH_3_L, ZnH_2_L, ZnHL, ZnL, and ZnH_–1_L (Table 2 and Figure 2B). The first detected complex species, ZnH_5_L, reaches its maximum concentration at pH ∼4.5 [∼60% of Zn(II) ions]. The coordinating environment of the metal ion at this pH is most probably two sulfur atoms derived from the cysteine thiol groups.^41,42^ In this complex species, Asp and Glu residues are also deprotonated. The coordination sphere of Zn(II) is most probably complemented by water molecules. The next detected complex form is ZnH_3_L [maximum concentration at pH 5.8; ∼80% of Zn(II) ions]. Most probably, it comes from the deprotonation of one cysteine and one histidine residue that could both be involved in Zn(II) binding. The next species, ZnH_2_L, reaches its maximum concentration at pH ∼8.0. It comes from the deprotonation of the second histidine residue. The p*K*_a_ value of this step equals 6.25 and is not significantly reduced compared to the p*K*_a_ of 6.74 for this residue in the free ligand, suggesting the nonbinding character of this residue. The Cys and His binding character was observed in the ^1^H–^1^H TOCSY spectrum of the Zn(II)–L680 system recorded at pH 7.4 (maximum of ZnH_2_L) (Figure 3B). The overlaid Hα–Hβ correlations of His and overlaid Hα–Hβ correlations of Cys are significantly affected by the presence of Zn(II) ions compared to the spectrum of the free ligand. These observations confirm that cysteine residues are mostly involved in Zn(II) binding, whereas at least one histidine residue strongly interacts with Zn(II). The shift of Hα–Hβ Ans-26 (nonbinding amino acid residue) is related to the close presence of metal ion. Another two detected complex species are ZnHL and ZnL, reaching their maximum concentrations at pH ∼8.0 and ∼10.0, respectively. The p*K*_a_ values of these steps equal 10.34 and 10.52, respectively; they arise from the deprotonation of two lysine residues that do not bind the central metal ion. ZnH_–1_L is the last detected complex. It appears in the solution at pH 10.0. This formation is most probably related to the deprotonation of the water molecule coordinated to the Zn(II) ion.

### Cd(II) Complexes

The results of mass spectrometry analysis confirmed the stoichiometry of the formed metal complexes. The mass spectra of the Cd(II)–L680 (charge of 3+) and Cd(II)–L681 (charge of 3+) systems are shown in Figures S2 and S3, respectively.

Both of the spectra show peaks corresponding to the free ligand ions ([L]^3+^, *m*/*z* 699.93 for L680 and *m*/*z* 719.92 for L681; *z* = 3+) and an equimolar complex with Cd(II) ions [[CdL]^3+^, *m*/*z* 737.27 for Cd(II)–L680 and *m*/*z* 756.92 for Cd(II)–L681; *z* = 3+]. In the middle of Figures S2 and S3, we can see the isotopic distribution of the mononuclear ligand–Cd(II) complex ion [CdL]^3+^. The 1:1 metal–ligand interaction is confirmed by potentiometric calculations.

Potentiometric titrations revealed that Cd(II)-Ac-_10_ACMECVRGHRSSSCKHHE_27_-NH_2_ system showed the existence of six complex forms at pH 2–11: CdH_5_L, CdH_4_L, CdH_3_L, CdH_2_L, CdHL, and CdL (Table 2 and Figure 4A). The first two detected complex species were CdH_5_L and CdH_4_L (maximum concentrations observed at pH ∼4.0 and ∼4.5, respectively). Most probably, they come from the deprotonation of three Cys residues^41^ and Glu residues are also deprotonated in these forms. A p*K*_a_ value of 4.12 is significantly reduced compared to p*K*_a_ values of 9.69, 8.68, and 8.00 for Cys residues in the free ligand, suggesting the presence of three Cys side chains in the coordination sphere of Cd(II). In the absorption spectrum of the Cd(II)–L680 system at pH 4.0–9.0 (Figure 5A), a characteristic band at ∼245 nm, commonly found in Cd(II)–peptide complexes, is discerned.^42^ It corresponds to the S^–^ to Cd(II) ligand to metal charge transfer transition and confirms the Cd(II) binding by Cys residues. At pH 4.0 (maximum concentration of CdH_5_L species), the intensity of a band increases significantly, which confirms that two Cys residues are involved in Cd(II) binding (Figure 5A). The increase in absorption intensity at pH 4.5 indicates the participation of the third Cys residue in the coordination sphere, which is in good agreement with the potentiometric results. No further significant increase in a band is observed at higher pH values, suggesting that all three Cys residues have already deprotonated and bind Cd(II). The next three detected complex forms are CdH_3_L, CdH_2_L, and CdHL (maximum concentrations observed at pH ∼5.0, ∼6.0, and ∼8.0, respectively). They arise from the deprotonation of three His residues that are not likely to bind Cd(II). NMR analysis of the Cd(II)–L680 system recorded at pH 7.4 revealed that all overlaid Cys Hα–Hβ correlations are significantly shifted due to strong interactions with Cd(II), whereas overlaid Hα–Hβ His-18, -25, and -26 correlations are only slightly broadened. This indicates that the coordination sphere of the metal ion consists of three Cys residues (Figure 6A). Moreover, the Glu Hα–Hβ correlations are significantly shifted, suggesting the proximity of the Cd(II) ion and the stabilizing role of these residues in the complex. The CdL is the last detected complex. It appears in the solution at pH 8.0. This formation is most probably related to the deprotonation lysine residue (the p*K*_a_ of this step equals 10.04), which is not involved in metal binding.

Peptide Ac-_10_ACDSCIKSHKAAQCEHNDR_28_-NH_2_ forms five complex species with Cd(II) ions at pH 2–11: CdH_5_L, CdH_4_L, CdH_3_L, CdH_2_L, and CdL (Table 2 and Figure 4B). The first two forms, CdH_5_L and CdH_4_L (their maximum concentrations are observed at pH ∼4 and ∼5, respectively), most probably come from the deprotonation of three Cys residues. Glu and Asp residues are also deprotonated in these species, but they are not likely to bind Cd(II). A p*K*_a_ value of 4.69 (linked with CdH_4_L formation) is significantly reduced compared to p*K*_a_ values of 9.42, 8.68, and 8.01 for Cys residues in the free ligand, suggesting the presence of three Cys side chains in the coordination sphere of Cd(II). The formation of appropriate complex forms was confirmed by UV–vis analysis. In the absorption spectrum of the Cd(II)–L681 system at pH 4.0–9.0 (Figure 5B), a band at ∼245 nm, characteristic of Cd(II)–peptide complexes, is observed.^42^ This corresponds to the S^–^ to Cd(II) charge transfer and confirms the Cd(II) binding by Cys residues. The maximum intensity of the absorption band was reached at pH 5.10 (maximum concentration of CdH_4_L species) (Figures 4B and 5B), which means that at this pH all three Cys residues coordinate to the Cd(II) ion.

The next two species, CdH_3_L and CdH_2_L, reach their maximum concentrations at pH ∼5.8 and ∼8.5, respectively. The p*K*_a_ values of these steps equal 5.19 and 6.42 and come from the deprotonation of the two histidine residues. Only a p*K*_a_ value of 5.19 is slightly reduced compared to those of His residues in the free ligand (5.96 and 6.74); however, histidine residues are not likely to bind Cd(II) ion. NMR analysis of the Cd(II)–L681 system recorded at pH 7.4 revealed that all Cys Hα–Hβ correlations are significantly shifted due to strong interactions with Cd(II), and we can also observe a slight shift of His and Asn-26 (neighbor of His-25) Hα–Hβ correlations (Figure 6B). As opposed to the Cd(II)–L680 system spectra, we did not detect a shift in Asp/Glu correlation signals. The next detected complex species was CdL, starting to form at pH 9.0. It arises from the deprotonation of two lysine residues that are not involved in metal binding.

**Figure 7:**
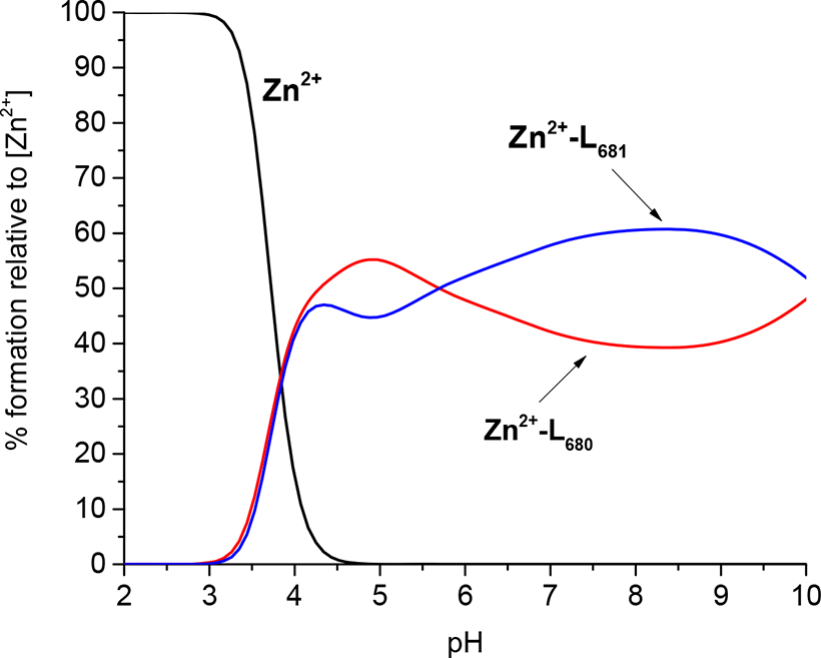
Competition plot between L680 and L681 ligand complexes with Zn(II) ion, showing complex formation in a hypothetical solution where metal ion and two peptides are present. The calculations based on the potentiometric data for studied systems are listed in Table 2.

The difference in the thermodynamic stability of the studied complexes can be shown in a competition plot, a chart based on the calculated stability constants. Competition plots show a hypothetical situation in which equimolar concentrations of all reagents are present. They are theoretical, based on potentiometric data. The plot reveals a higher stability of L681–Zn(II) than of L680–Zn(II) complexes above pH 5.8 (Figure 7). At pH >5.8 in both complexes, three Cys and His residues bind metal ion, but the interaction of Zn(II) with L680 amino acid residues is weaker than in the case of L681. This could be also observed in the NMR spectra (Figure 3) through a much smaller effect on Hα–Hβ His and Cys correlations of L680 in comparison to L681 after the addition of Zn(II) ions. This indicates that the interaction between L681 and Zn(II) is stronger and explains the difference in the stability of Zn(II)–L680 and Zn(II)–L681 systems over a wide pH range in the competition plot (Figure 7).

**Figure 8:**
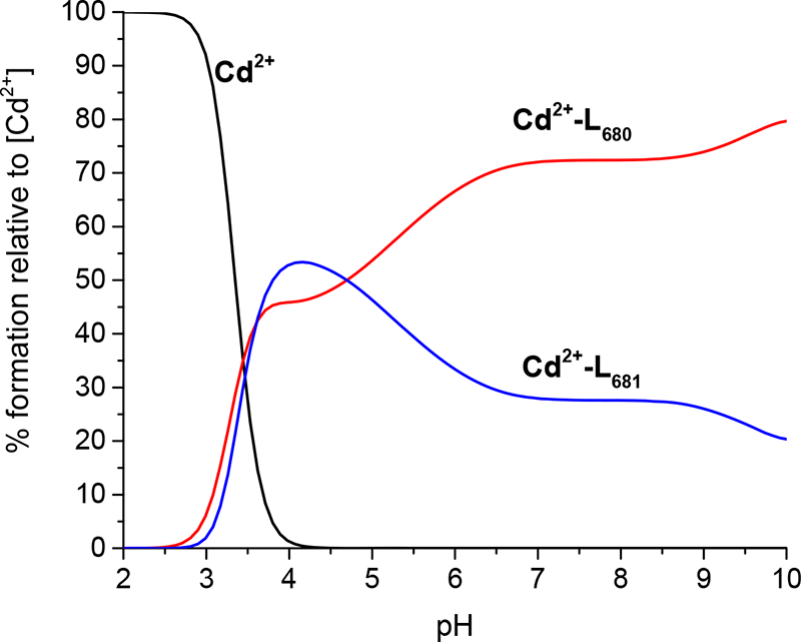
Competition plot between L680 and L681 ligand complexes with Cd(II) ion, showing complex formation in a hypothetical solution in which the metal ion and two peptides are present. The calculation based on the potentiometric data for the studied systems is given in Table 2.

The difference in the thermodynamic stability of the discussed Cd(II) complexes is shown in a competition plot (Figure 8). This reveals (i) a very similar stability of Cd(II)–L681 and Cd(II)–L680 complexes at pH 3.5–5.0 and (ii) a higher stability of Cd(II)–L680 than of Cd(II)–L681 complexes at pH >5.0. Interestingly, almost the reverse relationship is observed in the case of Zn(II) complexes. This suggests that L680 is for some reason a better ligand for soft Lewis acids, such as Cd(II), than L681, and L681 is a good donor for harder acids such as Zn(II), a borderline Lewis acid (intermediate). At pH >5.0, the Cd(II)–L680 system is more stable than the Cd(II)–L681 system due to a strong interaction of Cd(II) ions with glutamic acid residues. Glutamic acid residues are not likely to bind the Cd(II) ion but can stabilize the structure of a complex.^43^ However, this phenomenon of a stabilizing effect was not observed for His residues. This result is in agreement with NMR analysis. We observed the shift of the Hα–Hβ Glu correlations in the spectra of the Cd(II)–L680 system, confirming the impact of Glu residues on the stability of the Cd(II)–L680 system.

**Figure 9:**
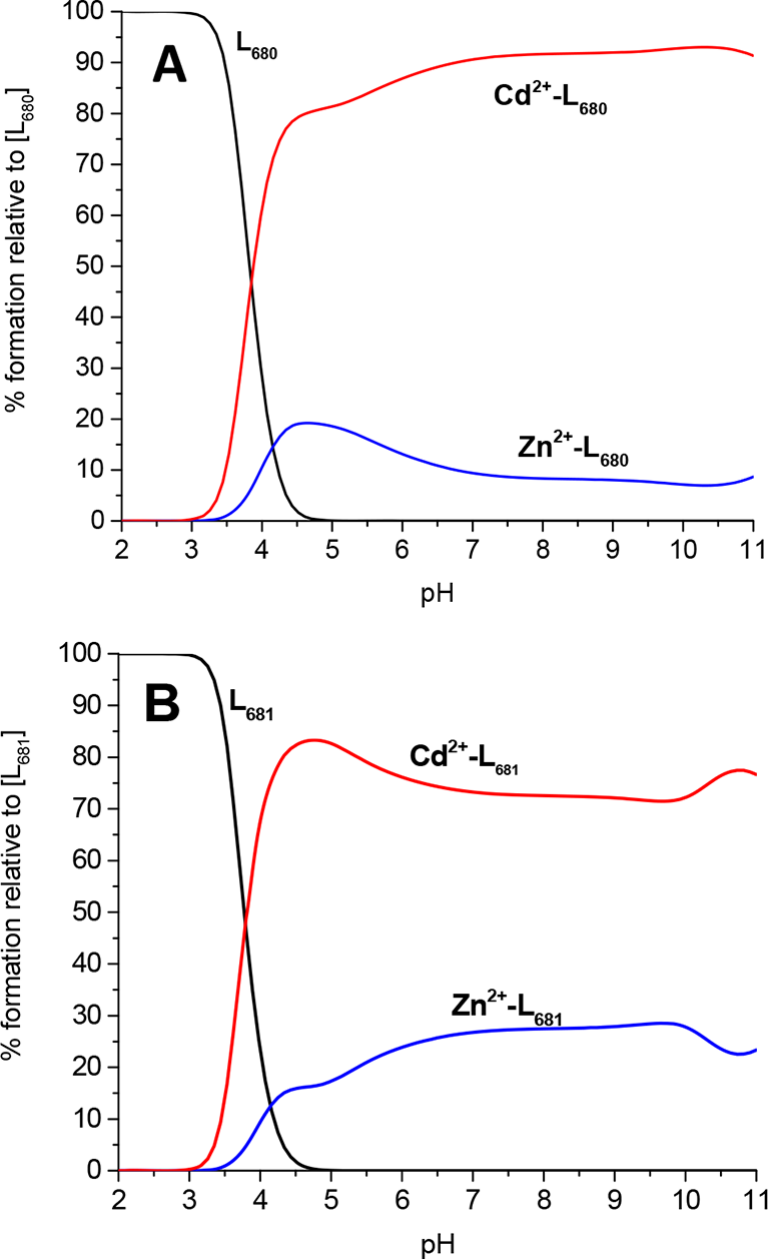
Competition plots between Zn(II) and Cd(II) ions and ligands (A) L680 and (B) L681 showing complex formation in a hypothetical solution in which two metal ions and the peptide are present. The calculation based on the potentiometric data for studied systems is shown in Table 2.

To establish the affinity of examined peptides for Cd(II) and Zn(II) ions, we calculated competition plots showing the stability of L681 and L680 complex systems with examined metals (Figure 9A,B). It is clear that both ligands create significantly more stable complex systems with Cd(II) ions than with Zn(II). This is due to the character of electron donors (Lewis bases) and acceptors (Lewis acids) in coordination mode. In Pearson theory, soft acids interact strongly with soft bases. Cysteine residues, the most important donors of electron density in examined complexes, act as soft bases due to the relatively large size of their thiol groups. The ion radius of Cd(II) ions is larger than that of Zn(II) due to the larger number of electron shells, and it acts more like a strong Lewis acid. Therefore, the Cd(II)–Cys3-type complex is more stable than the Zn(II)–Cys3 complex.

According to the potentiometric and MS results, both model peptides form equimolar complexes with Zn(II) and Cd(II). Another similarity is that in L680 and L681 Cys and His residues are involved in direct Zn(II) binding, which was confirmed by NMR analysis. All three Cys residues bind the metal ion in L680 and L681 complexes. The correlation signals of His residues overlapped, which hampered the determination of binding sites. According to the UniProt database, His-25 of L681/L680 should be involved in Zn(II) binding by analogy to the Cys_3_His-type zinc finger structure of other known yeast metal-regulated transcription factors.^26−30^ Moreover, this site in studied structures is the same, while His-26 is present in only the L680 ligand. It is more likely that the nature would create a binding motif that can be repeated in many proteins. His-18 is common for both of the proteins; however, it could not be used as a Zn(II)-binding site probably because its position makes it impossible for the metal ion to form a complex with a favorable geometry. These results show that in the isolated fragments the Zn(II)-binding motif remains the same and that studied peptides are reliable models of MAC1/AMT1 behavior in the presence of metal ions. More interestingly, we have shown that even nonbinding histidine residues play a significant role in providing complex stability: L681 forms a more stable Zn(II) complex due to a strong stabilizing effect of His-18, which was confirmed by NMR analysis.

**Figure 10:**
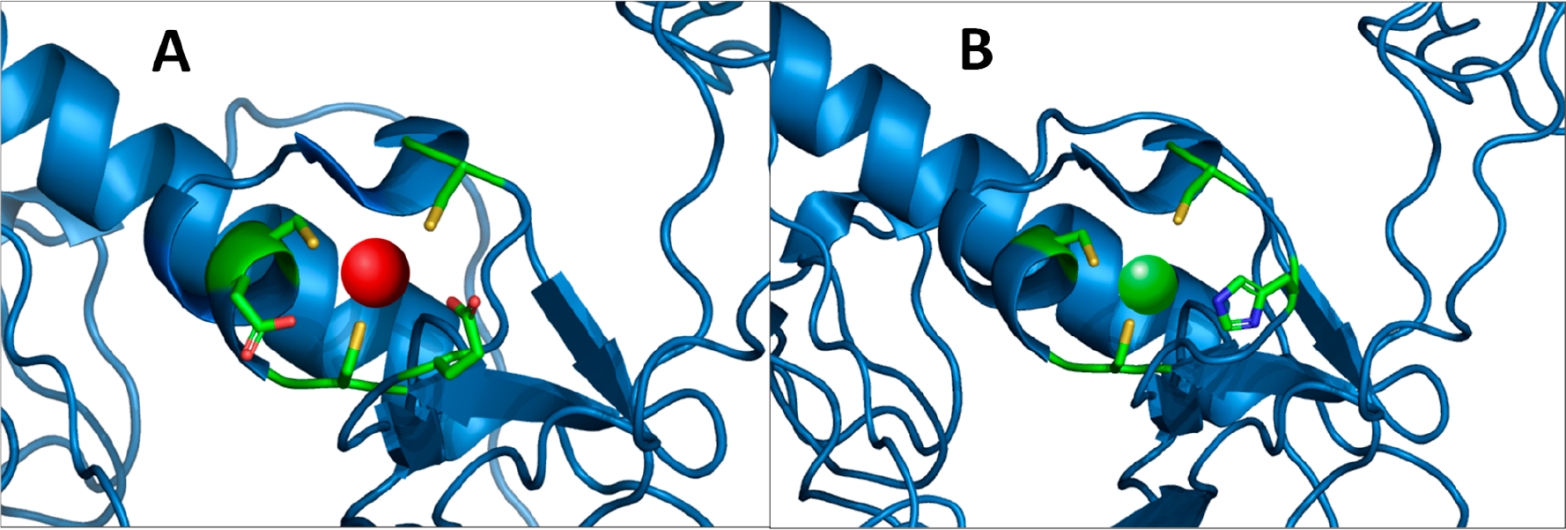
Models of (A) Cd(II)–MAC1 and (B) Zn(II)–MAC1 complexes. The structure of the MAC1 is based on simulation by Phyre2. Figures were generated using PyMOL software.

The studies of Cd(II)–L systems produced even more interesting results. We have not only confirmed the binding sites of studied ligands (all three Cys residues present in L680 and L681) but also noticed the role of amino acid residues adjacent to cysteine and histidine residues on the stability of complexes. We acknowledged that the data obtained for Cd(II)–L systems are quite different from those of Zn(II)–L systems. According to the analysis of the NMR spectra, in Cd(II)–L systems only cysteine residues bind the metal ion, while in the Zn(II)–L system, histidine residues are also involved in metal binding. This is in agreement with the literature data^43^ and is the first difference between Zn(II) and Cd(II) complexes. We have also acknowledged that different correlation signals of the same ligand [e.g., His/Glu in the case of the Cd(II)–L680 system] were affected by the presence of Zn(II) and Cd(II), except cysteine residues. The comparison of the NMR spectra “reveals the identity” of Cd(II) in the studied systems. We summed up our results in the form of graphical models pictured in Figure 10. This led us to two conclusions. (i) In our studies, Cd(II) is a good Zn(II) probe for investigating the stoichiometry of complexes but not exactly the binding sites and stability. (ii) The presence of amino acid residues such as Glu in a cysteine-rich domain may stabilize the structure of some Cd(II) complexes (like in the case of L680), but not all (like in the case of L681). The last discovery is in agreement with the literature, which shows the examples of Cd(II)–peptide complexes stabilized by the presence of a Glu/Asp residue. Indeed, the shifts of Glu correlations in the Cd(II)–L680 system are significant and were not observed in the case of the Cd(II)–L681 system, which suggests the involvement of these amino acid residues in providing the complex stability. We can observe these phenomena in the competition plot (Figure 8). These results present interesting properties of zinc finger domains of fungal MAC1 and AMT1 as ligands for metal ions. They also indicate that (i) the Glu residue stabilizes the structure of the Cd(II)–L680 complex, but not the Cd(II)–L681 complex, and (ii) Cd(II) “replaces” but does not exactly “mimic” Zn(II) in investigated L680 and L681 systems. The explanation of the first (i) phenomenon could be as follows. In L680 (Ac-_10_ACMECVRGHRSSSCKHHE_27_-NH_2_) actually two Glu residues are present in the sequence; therefore, the stabilizing effect should be stronger. A more detailed explanation could concern the position of appropriate Glu residues in the peptide sequence. In L680, one of the Glu residues is located between two binding Cys residues, so the distance between Cd(II) and Glu is short and allows for strong interaction. In L681 (Ac-_10_ACDSCIKSHKAAQCEHNDR_28_-NH_2_), the only Glu residue is located near one binding Cys residue that could increase the distance between Cd(II) and this particular Glu residue and therefore weaken the possible interaction. This phenomenon may be also an explanation of why L680 forms more stable complexes with Zn(II) and L681 with Cd(II) when competing with the other ligand (Figures 7 and 8). In the case of Zn(II) complexes, we did not observe the impact of Glu residues on stability; meanwhile, in the case of Cd(II) complexes, the presence of Glu in the appropriate position of the sequence significantly supports the stability of the complexes. The explanation of the second (ii) result should be more nuanced and tricky. Cd(II)–peptide systems are still not well described in the literature. However, there are some examples of protein systems in which Cd(II) is successfully used to study particular properties of Zn(II)-binding proteins. It is a very sufficient approach to picture a whole system and focus on more general problems, e.g., the arrangement of protein domains around the metal ion. In our case, very particular, detailed, and specific domains were studied, and we focused on the comparison of two similar domains as ligands for Zn(II) and Cd(II). We have shown that even very similar ligands may differ from the coordination chemistry point of view. We could say that in the case of particular systems studied in this research, Cd(II) “replaces” but does not exactly “mimic” Zn(II).

## Conclusions

In this work, we present interesting features of MAC1 and AMT1 zinc finger domains as ligands for Zn(II) and Cd(II) ions. We confirmed that all studied complexes are characterized by equimolar stoichiometry. The set of donors for Cd(II) and Zn(II) complexes is different. In Zn(II)–L systems, both Cys and His residues are involved in metal binding, and in Cd(II)–L systems, only Cys residues are involved. When competing for the ligand, Cd(II) obviously wins with Zn(II) due to the “soft” character of this metal ion according to Pearson’s theory. We found, however, some interesting differences in complex stability. (i) L681 forms more stable complexes with Zn(II) than L680 due to the supportive role of histidine residues. (ii) L680 forms more stable complexes with Cd(II) than L681 as a result of strong interactions with a nonbinding Glu residue. What is more relevant is that the comparison of Zn(II) and Cd(II) systems for the same ligand allowed the observation of significant differences concerning preferable metal-binding sites and the supporting impact of other amino acid residues on complex stability. This led us to conclude that in the case of studied peptides Cd(II) is not exactly able to “mimic zinc”.

## Supporting Information Available

Complementary mass spectra (Figures S1–S3) (PDF)

## References

[ref1] Prasad R.; Banerjee A.; Khandelwal N. K.; Dhamgaye S. The ABCs of Candida albicans Multidrug Transporter Cdr1. Eukaryotic Cell 2015, 14 (12), 1154–1164. 10.1128/EC.00137-15. 26407965 PMC4664872

[ref2] Colombo A. L.; Júnior J. N. de A.; Guinea J. Emerging multidrug-resistant Candida species. Current Opinion in Infectious Diseases 2017, 30 (6), 528–538. 10.1097/QCO.0000000000000411. 29095200

[ref3] Arendrup M. C.; Patterson T. F. Multidrug-Resistant Candida: Epidemiology, Molecular Mechanisms, and Treatment. J. Infect. Dis. 2017, 216, S445–S451. 10.1093/infdis/jix131. 28911043

[ref4] Healey K. R.; Jimenez Ortigosa C.; Shor E.; Perlin D. S. Genetic Drivers of Multidrug Resistance in Candida glabrata. Frontiers in Microbiology 2016, 7, 1–9. 10.3389/fmicb.2016.01995. 28018323 PMC5156712

[ref5] Sanglard D.; Odds F. C. Resistance of Candida species to antifungal agents: molecular mechanisms and clinical consequences. Lancet Infect Dis 2002, 2, 73–85. 10.1016/S1473-3099(02)00181-0. 11901654

[ref6] Brown G. D.; Denning D. W.; Levitz S. M. Tackling human fungal infections. Science 2012, 336, 647 10.1126/science.1222236. 22582229

[ref7] Fox E. P.; Nobile C. J. The role of *Candida albicans* biofilms in human disease. In Candida albicans symptoms, causes and treatment options; Dietrich L. A., Friedmann T. S., Eds.; Nova Science Publishers, 2013; pp 1–24.

[ref8] Douglas L. J. *Candida* biofilms and their role in infection. Trends Microbiol 2003, 11, 30–6. 10.1016/S0966-842X(02)00002-1. 12526852

[ref9] Gudlaugsson O.; Gillespie S.; Lee K.; Van de Berg J.; Hu J.; Messer S.; Herwaldt L.; Pfaller M.; Diekema D. Attributable mortality of nosocomial candidemia, revisited. Clin. Infect. Dis. 2003, 37, 1172–1177. 10.1086/378745. 14557960

[ref10] Wisplinghoff H.; Bischoff T.; Tallent S. M.; Seifert H.; Wenzel R. P.; Edmond M. B. Nosocomial bloodstream infections in US hospitals: analysis of 24,179 cases from a prospective nationwide surveillance study. Clin. Infect. Dis 2004, 39, 309–317. 10.1086/421946. 15306996

[ref11] Nobile C. J.; Johnson A. D. *Candida albicans* biofilms and human disease. Annu. Rev. Microbiol. 2015, 69, 71–92. 10.1146/annurev-micro-091014-104330. 26488273 PMC4930275

[ref12] Chandra J.; Kuhn D. M.; Mukherjee P. K.; Hoyer L. L.; McCormick T.; Ghannoum M. A. Biofilm formation by the fungal pathogen *Candida albicans*: development, architecture, and drug resistance. J. Bacteriol. 2001, 183, 5385–94. 10.1128/JB.183.18.5385-5394.2001. 11514524 PMC95423

[ref13] Gulati M.; Nobile C. J. Candida albicans biofilms: development, regulation, and molecular mechanisms. Microbes and Infection 2016, 18 (5), 310–321. 10.1016/j.micinf.2016.01.002. 26806384 PMC4860025

[ref14] Fox E. P.; Singh-babak S. D.; Hartooni N.; Nobile C. J. Biofilms and antifungal resistance. In Antifungals from genomics to resistance and the development of novel agents; Coste A. T., Vandeputte P., Eds.; Caister Academic Press, 2015; pp 71–90. 10.21775/9781910190012

[ref15] Andes D. R.; Safdar N.; Baddley J. W.; Playford G.; Reboli A. C.; Rex J. H.; Sobel J. D.; Pappas P. G.; Kullberg B. J. Mycoses Study Group. Impact of treatment strategy on outcomes in patients with candidemia and other forms of invasive candidiasis: a patient-level quantitative review of randomized trials. Clin Infect Dis 2012, 54, 1110–22. 10.1093/cid/cis021. 22412055

[ref16] Hajjeh R. A.; Sofair A. N.; Harrison L. H.; Lyon G. M.; Arthington-Skaggs B. A.; Mirza S. A.; Phelan M.; Morgan J.; Lee-Yang W.; Ciblak M. A.; Benjamin L. A.; Thomson Sanza L.; Huie S.; Fah Yeo S.; Brandt M. E.; Warnock D. W. Incidence of bloodstream infections due to *Candida* species and *in vitro* susceptibilities of isolates collected from 1998 to 2000 in a population-based active surveillance program. J. Clin. Microbiol. 2004, 42, 1519–1527. 10.1128/JCM.42.4.1519-1527.2004. 15070998 PMC387610

[ref17] Pfaller M. A.; Moet G. J.; Messer S. A.; Jones R. N.; Castanheira M. *Candida* bloodstream infections: comparison of species distributions and antifungal resistance patterns in Community-onset and nosocomial isolates in the SENTRY Antimicrobial Surveillance Program, 2008–2009. Antimicrob. Agents Chemother. 2011, 55, 561–566. 10.1128/AAC.01079-10. 21115790 PMC3028787

[ref18] Sanglard D.; Ischer F.; Calabrese D.; Majcherczyk P. A.; Bille J. The ATP binding cassette transporter gene CgCDR1 from *Candida glabrata* is involved in the resistance of clinical isolates to azole antifungal agents. Antimicrob. Agents Chemother. 1999, 43, 2753–2765. 10.1128/AAC.43.11.2753. 10543759 PMC89555

[ref19] Izumikawa K.; Kakeya H.; Tsai H. F.; Grimberg B.; Bennett J. E. Function of *Candida glabrata* ABC transporter gene, PDH1. Yeast 2003, 20, 249–261. 10.1002/yea.962. 12557277

[ref20] Li C. X.; Gleason J. E.; Zhang S. X.; Bruno V. M.; Cormack B. P.; Culotta V. C. Candida albicans adapts to host copper during infection by swapping metal cofactors for superoxide dismutase. Proc. Natl. Acad. Sci. U. S. A. 2015, 112 (38), E5336–E5342. 10.1073/pnas.1513447112. 26351691 PMC4586888

[ref21] Gerwien F.; Skrahina V.; Kasper L.; Hube B.; Brunke S. Metals in fungal virulence. FEMS Microbiol. Rev. 2017, 42 (1), fux050 10.1093/femsre/fux050. PMC5812535 29069482

[ref22] Turner R. B.; Smith D. L.; Zawrotny M. E.; Summers M. F.; Posewitz M. C.; Winge D. R. Solution structure of a zinc domain conserved in yeast copper-regulated transcription factors. Nat. Struct. Biol. 1998, 5 (7), 551–555. 10.1038/805. 9665167

[ref23] Marvin M. E.; Mason R. P.; Cashmore A. M. The CaCTR1 gene is required for high-affinity iron uptake and is transcriptionally controlled by a copper-sensing transactivator encoded by CaMAC1. Microbiology 2004, 150 (7), 2197–2208. 10.1099/mic.0.27004-0. 15256562

[ref24] Huang G.-H.; Nie X.-Y.; CHEN J.-Y. CaMac1, a Candida albicans Copper Ion-sensing Transcription Factor, Promotes Filamentous and Invasive Growth in Saccharomyces cerevisiae. Acta Biochimica et Biophysica Sinica 2006, 38 (3), 213–217. 10.1111/j.1745-7270.2006.00146.x. 16518547

[ref25] Hauser N. C.; Dukalska M.; Fellenberg K.; Rupp S. From experimental setup to data analysis in transcriptomics: copper metabolism in the human pathogen Candida albicans. Journal of Biophotonics 2009, 2 (4), 262–268. 10.1002/jbio.200910004. 19367594

[ref26] https://www.uniprot.org/uniprotkb/P15315/entry#family_and_domains (accessed 2022-07-27).

[ref27] https://www.uniprot.org/uniprotkb/P35192/entry#family_and_domains (accessed 2022-07-27).

[ref28] https://www.uniprot.org/uniprotkb/Q12753/entry#family_and_domains (accessed 7/27/2022-07-27).

[ref29] https://www.uniprot.org/uniprotkb/P45815/entry#family_and_domains (accessed 7/27/2022-07-27).

[ref30] https://www.uniprot.org/uniprotkb/Q92258/entry#family_and_domains (accessed 7/27/2022-07-27).

[ref31] Staats C. C.; Kmetzsch L.; Schrank A.; Vainstein M. H. Fungal zinc metabolism and its connections to virulence. Front. Cell. Infect. Microbiol. 2013, 3, 65 10.3389/fcimb.2013.00065. 24133658 PMC3796257

[ref32] Trevors J. T.; Stratton G. W.; Gadd G. M. Cadmium transport, resistance, and toxicity in bacteria, algae, and fungi. Can. J. Microbiol. 1986, 32 (6), 447–464. 10.1139/m86-085. 3089567

[ref33] Funk A. E.; Day F. A.; Brady F. O. Displacement of zinc and copper from copper-induced metallothionein by cadmium and by mercury: in vivo and ex vivo studies. Comp. Biochem. Physiol., Part C: Toxicol. Pharmacol. 1987, 86 (1), 1–6. 10.1016/0742-8413(87)90133-2. 2881702

[ref34] Day F. A.; Funk A. E.; Brady F. O. In vivo and ex vivo displacement of zinc from metallothionein by cadmium and by mercury. Chem. Biol. Interact 1984, 50 (2), 159–74. 10.1016/0009-2797(84)90093-0. 6744462

[ref35] Hasler D. W.; Faller P.; Vašák M. Metal–Thiolate Clusters in the C-Terminal Domain of Human Neuronal Growth Inhibitory Factor (GIF). Biochemistry 1998, 37 (42), 14966–14973. 10.1021/bi9813734. 9778374

[ref36] Armitage I. M.; Drakenberg T.; Reilly B. Use of ^113^Cd NMR to Probe the Native Metal Binding Sites in Metalloproteins: An Overview. Coord. Chem. Rev. 1988, 11, 117–144. 10.1007/978-94-007-5179-8_6. PMC5245840 23430773

[ref37] Gran G.; Dahlenborg H.; Laurell S.; Rottenberg M. Determination of the equivalent point in potentiometric titrations. Acta Chem. Scand. 1950, 4 (4), 559–577. 10.3891/acta.chem.scand.04-0559.

[ref38] Gans P.; Sabatini A.; Vacca A. Investigation of equilibria in solution. Determination of equilibrium constants with the HYPERQUAD suite of programs. Talanta 1996, 43, 1739–1753. 10.1016/0039-9140(96)01958-3. 18966661

[ref39] Alderighi L.; Gans P.; Ienco A.; Peters D.; Sabatini A.; Vacca A. Hyperquad simulation and speciation (HySS): a utility program for the investigation of equilibria involving soluble and partially soluble species. Coord. Chem. Rev. 1999, 184, 311–318. 10.1016/S0010-8545(98)00260-4.

[ref40] Grimsley G. R.; Scholtz J. M.; Pace C. N. A summary of the measured pK values of the ionizable groups in folded proteins. Protein Sci. 2009, 18 (1), 247–251. 10.1002/pro.19. 19177368 PMC2708032

[ref41] Kolkowska P.; Krzywoszynska K.; Potocki S.; Chetana P. R.; Spodzieja M.; Rodziewicz-Motowidlo S.; Kozlowski H. Specificity of the Zn2+, Cd2+ and Ni2+ ion binding sites in the loop domain of the HypA protein. Dalton Transactions 2015, 44 (21), 9887–9900. 10.1039/C5DT01005E. 25945782

[ref42] Potocki S.; Rowinska-Zyrek M.; Valensin D.; Krzywoszynska K.; Witkowska D.; Luczkowski M.; Kozlowski H. Metal Binding Ability of Cysteine-Rich Peptide Domain of ZIP13 Zn2+Ions Transporter. Inorg. Chem. 2011, 50 (13), 6135–6145. 10.1021/ic200270p. 21630642

[ref43] Sóvágó I.; Várnagy K. Cadmium(II) Complexes of Amino Acids and Peptides. Met. Ions Life Sci. 2012, 275–302. 10.1007/978-94-007-5179-8_9. 23430776

