# Supplementary material for: Zn(II) and Cd(II) Complexes of AMT1/MAC1 Homologous Cys/His-Rich Domains: So Similar yet So Different
Source: Inorg Chem. 2022 Aug 31;61(36):14333–43. doi: 10.1021/acs.inorgchem.2c02080 (PMC9472267; doi:10.1021/acs.inorgchem.2c02080)
Supplement: Supplementary file 1 — ic2c02080_si_001.pdf [file ic2c02080_si_001.pdf]

Zn(II) and Cd(II) Complexes of AMT1/MAC1 Homologous Cys/His-Rich Domains - So Similar yet So Different

Anna Rola,[1] Paulina Potok,[1] Magdalena Mos,[2] Elżbieta Gumienna-Kontecka,[1] Sławomir Potocki[1*]

slawomir.potocki@chem.uni.wroc.pl

[1] Faculty of Chemistry, University of Wroclaw, 14 Joliot-Curie Street, 50-383 Wroclaw, Poland

[2] WMG, International Manufacturing Centre, University of Warwick, Coventry CV4 7AL, United Kingdom

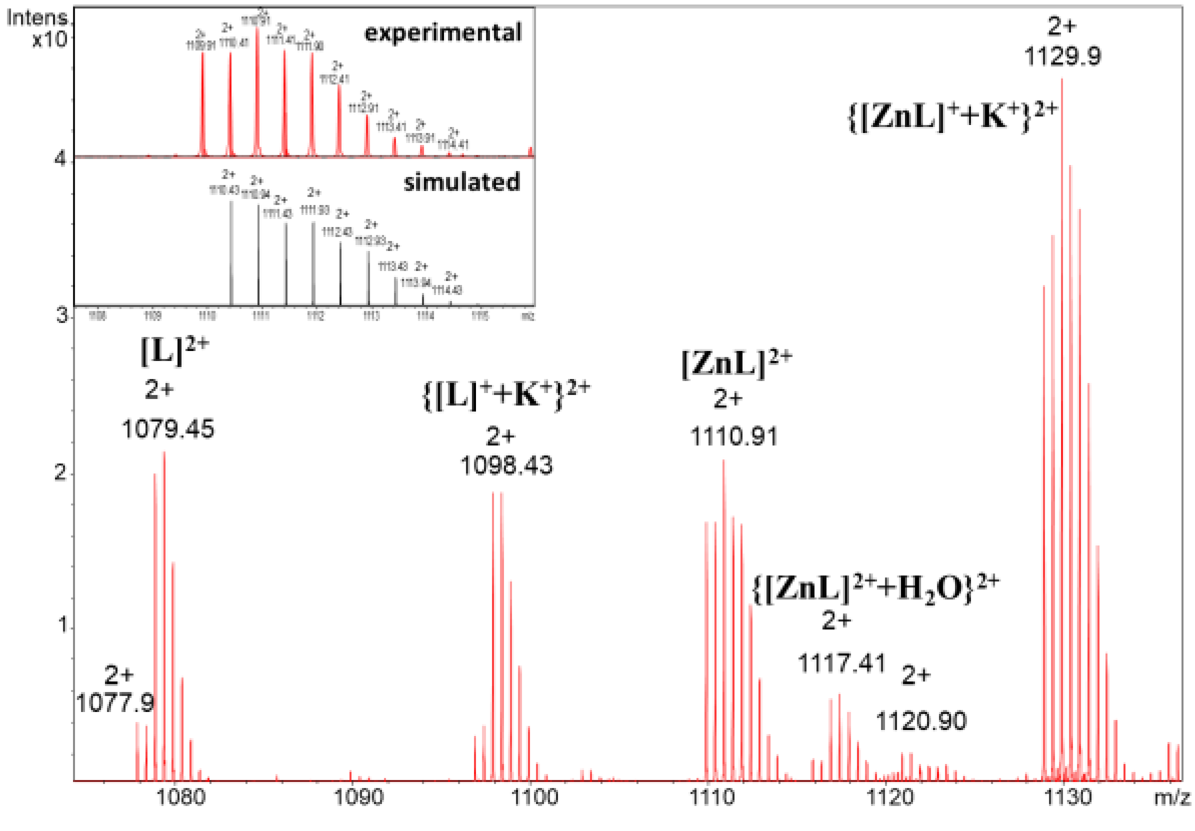

Fig. S1 ESI-MS spectrum of a system composed of the Ac-$_{10}$ACDSCIKSHKAAQCEHNDR$_{28}$-NH_2 ligand (L681) and Zn(II) ions in the range of *m/z* 1075–1135 at pH 7.4 (1:1 M:L). In the top left corner, the simulated and experimental isotopic distribution spectra with a peak at *m/z* 1110.91 are presented.

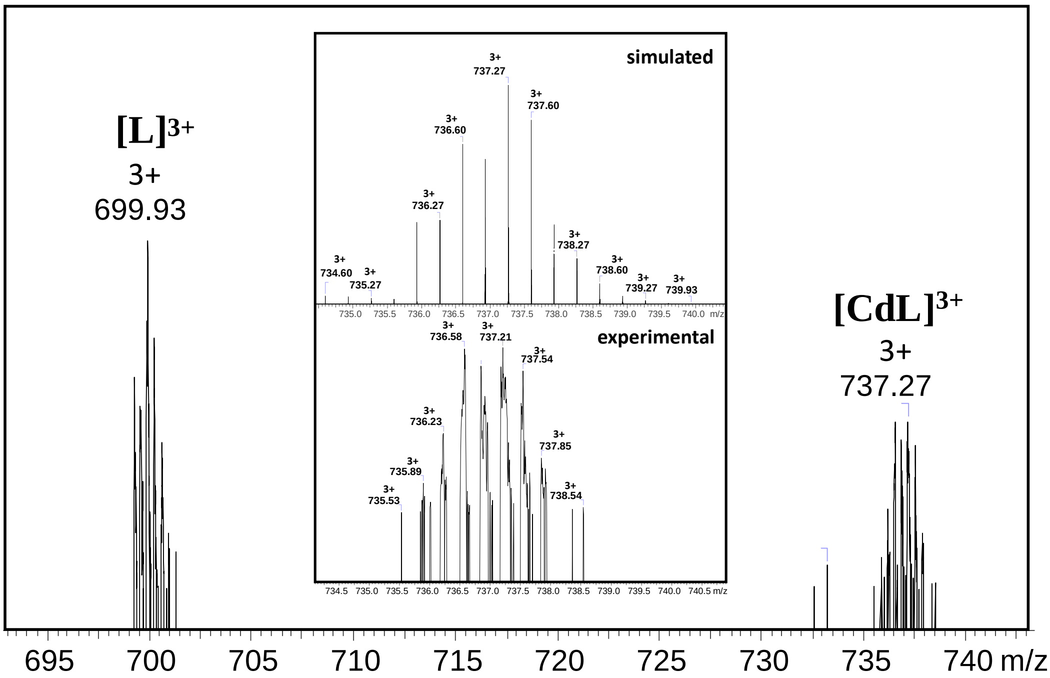

Fig. S2 ESI-MS spectrum of a system composed of the Ac-$_{10}$ACMECVRGHRSSSCKHHE$_{27}$ ligand (L680) and Cd(II) ions in the range of *m/z* 690–750 at pH 7.4 (1:1 M:L). In the middle, the simulated and experimental isotopic distribution spectra with a peak at *m/z* = 737.27 are presented.

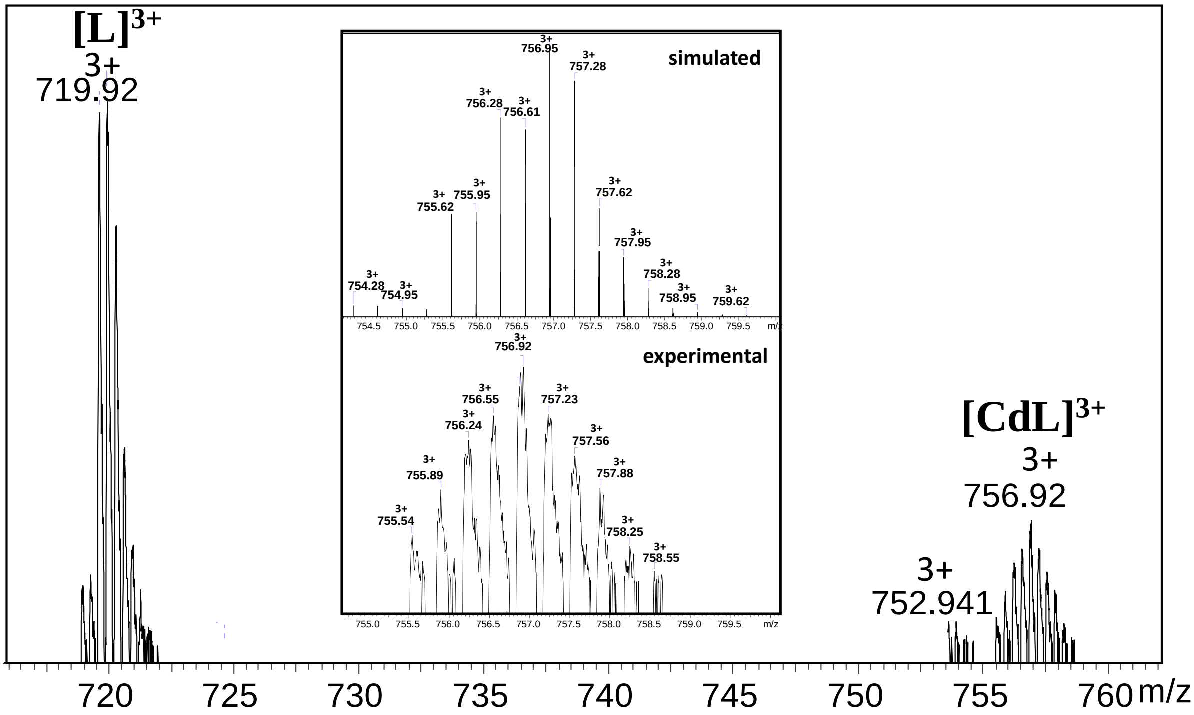

Fig. S3 ESI-MS spectrum of a system composed of the Ac-$_{10}$ACDSCIKSHKAAQCEHNDR$_{28}$-NH$_2$ ligand (L681) and Cd(II) ions in the range of *m/z* 715-765 at pH 7.4 (1:1 M:L). In the middle, the simulated and experimental isotopic distribution spectra with a peak at *m/z* = 756.92 are presented.
